# Spliceosome-mediated decay (SMD) regulates expression of nonintronic genes in budding yeast

**DOI:** 10.1101/gad.221960.113

**Published:** 2013-09-15

**Authors:** Adam Volanakis, Monica Passoni, Ralph D. Hector, Sneha Shah, Cornelia Kilchert, Sander Granneman, Lidia Vasiljeva

**Affiliations:** 1 Department of Biochemistry, University of Oxford, Oxford OX1 3QU, United Kingdom;; 2 Institute for Structural and Molecular Biology, Centre for Synthetic and Systems Biology (SynthSys), University of Edinburgh, Edinburgh EH9 3JD, United Kingdom

**Keywords:** spliceosome, mRNA, spliceosome-mediated decay (SMD), exosome, RNA degradation

## Abstract

We uncovered a novel role for the spliceosome in regulating mRNA expression levels that involves splicing coupled to RNA decay, which we refer to as spliceosome-mediated decay (SMD). Our transcriptome-wide studies identified numerous transcripts that are not known to have introns but are spliced by the spliceosome at canonical splice sites in *Saccharomyces cerevisiae*. Products of SMD are primarily degraded by the nuclear RNA surveillance machinery. We demonstrate that SMD can significantly down-regulate mRNA levels; splicing at canonical splice sites in the bromodomain factor 2 (*BDF2*) transcript reduced transcript levels roughly threefold by generating unstable products that are rapidly degraded by the nuclear surveillance machinery. Regulation of *BDF2* mRNA levels by SMD requires Bdf1, a functionally redundant Bdf2 paralog that plays a role in recruiting the spliceosome to the *BDF2* mRNA. Interestingly, mutating *BDF2* 5′ splice site and branch point consensus sequences partially suppresses the *bdf1Δ* temperature-sensitive phenotype, suggesting that maintaining proper levels of Bdf2 via SMD is biologically important. We propose that the spliceosome can also repress protein-coding gene expression by promoting nuclear turnover of spliced RNA products and provide an insight for coordinated regulation of Bdf1 and Bdf2 levels in the cell.

## Abstract (precis)

This study uncovers a role for the spliceosome in regulating mRNA expression levels. Transcriptome-wide studies reveal splice junctions in transcripts that are not known to have introns in budding yeast. Volanakis et al. show that spliceosomal cleavage of bromodomain factor 2 (BDF2) mRNA generates unstable products degraded by the nuclear surveillance machinery, and BDF2 regulation requires its paralog, Bdf1. The authors thus propose a mechanism—termed spliceosome-mediated decay (SMD)—for the regulation of gene expression involving splicing coupled to RNA decay.

The biogenesis of pre-mRNA involves multiple processing reactions, including 5′ capping, splicing, cleavage, and polyadenylation at the 3′ end, leading to production of translationally competent mRNA (Moore and Proudfoot 2009). In nuclear pre-mRNA splicing, the excision of introns is catalyzed by the spliceosome, a ribonucleoprotein machine comprising five small nuclear RNAs (snRNAs) and >100 conserved proteins (Wahl et al. 2009). Spliceosomal snRNAs contain a conserved PuAU_4–6_GPu sequence called the Sm site, which provides a platform for the assembly of the heteroheptameric Sm complex, comprised of seven Sm proteins: SmB/B′, SmD1, SmD2, SmD3, SmE, SmF, and SmG (Beggs 2005). These form a ring, referred to as the Sm complex, around the Sm site of the spliceosomal U1, U2, U4, and U5 snRNAs, and play roles in multiple aspects of small nuclear ribonucleoprotein (snRNP) biogenesis. The U6 snRNA associates with a structurally related set of seven Lsm (like Sm) proteins. Being a part of several snRNPs, the Sm complex was shown to facilitate assembly of the spliceosome on pre-mRNA (Zhang et al. 2001) and play a role in multiple aspects of snRNA biogenesis, such as cellular localization, processing, and stability.

The spliceosomal snRNPs and multiple non-snRNP proteins assemble cotranscriptionally on pre-mRNAs through recognition of the 5′ splice site (5′ss), the branch point (BP), and the 3′ss to form the spliceosome (Carrillo Oesterreich et al. 2011). This involves interactions of the spliceosomal components with the 5′ cap-binding complex and the C-terminal domain (CTD) of RNA polymerase II (RNA Pol II). Splicing factors such as yeast Prp40 and several human SR proteins were implicated in mediating interactions with the phosphorylated CTD (Morris and Greenleaf 2000; Kotovic et al. 2003). Previous analyses of spliceosomal recruitment to RNA Pol II-transcribed genes were performed by studying the genome-wide distribution of the individual components of the spliceosome using chromatin immunoprecipitation (ChIP) (Kotovic et al. 2003; Moore et al. 2006; Tardiff et al. 2006). Surprisingly, these studies revealed that spliceosomal components are also recruited to some genes whose transcripts are not known to be spliced. It was recently shown that several nonintronic transcripts can be spliced; however, it remained unclear whether this had any regulatory role or was the result of stochastic splicing events that occur due to the lack of specificity in the spliceosome recruitment (Harigaya and Parker 2012).

Unexpectedly, by sequencing SmD1-associated RNAs, we identified many RNA Pol II transcripts that are not known to be spliced, indicating that spliceosome assembly on nonintronic mRNAs may be a common phenomenon. To understand the functional significance of this discovery, we analyzed the effect of splicing mutants and strains defective in RNA surveillance on the protein-coding transcriptome. Remarkably, these mutants displayed a marked up-regulation of a number of intronless mRNAs, including bromodomain factor 2 (*BDF2*) mRNA, which encodes a bromodomain factor, and *OYE3* mRNA, which encodes an NADPH oxidoreductase. Interestingly, it was previously shown that *BDF2* overexpression is toxic, implying that it could be important to down-regulate levels of this transcript in the cell (Yoshikawa et al. 2011; Fu et al. 2013). We demonstrate that splicing of these mRNAs at consensus splice sites generates unstable products that are primarily degraded by the nuclear RNA surveillance machinery. This spliceosome-mediated decay (hereafter referred to as SMD) of *BDF2* mRNA is dependent on Bdf1, another bromodomain-containing protein, providing a plausible explanation of how expression of these paralogous genes is regulated. Mutating *BDF2* 5′ss and BP consensus sequences suppressed *bdf1Δ* growth phenotypes, suggesting that maintaining proper levels of Bdf2 via SMD is biologically important. We propose a model in which SMD regulates coordinated expression of Bdf1 and Bdf2. Collectively, our results have revealed a new role for the spliceosome in the regulation of mRNA expression.

## Results

### Purification and analyses of the Sm complex-bound RNAs

To identify RNA targets of the spliceosome, we purified spliceosome-associated RNAs by affinity chromatography using a strain expressing tandem affinity purification (TAP)-tagged SmD1, a protein required for stabilizing the U1 snRNA–pre-mRNA interaction (Zhang et al. 2001). As a negative control, we used a nontagged parental strain. SmD1-coprecipitated RNA was extracted and analyzed by Northern blotting and high-throughput RNA sequencing (RNA-seq). Methylene blue staining of total RNA showed a significant depletion of ribosomal RNAs (rRNAs) in the SmD1 pull-down compared with input (Supplemental Fig. 1A). Conversely, Northern analysis revealed substantial enrichment of the Sm site-containing RNAs U1 (Supplemental Fig. 1A) and telomerase (*TLC1*) (data not shown), validating the biological relevance of this approach. U1 was undetectable in the mock samples, demonstrating the specificity of the results (Supplemental Fig. 1A, lanes 5,7). Mass spectrometric analysis of coprecipitated proteins identified other Sm complex members (SmB, SmD1, SmD2, SmE, and SmG) and many spliceosome components (Supplemental Fig. 1B).

Bioinformatics analysis of the RNA-seq data (Supplemental Table 1) revealed that 35%–40% of uniquely mapped cDNAs corresponded to snRNAs directly bound by the Sm complex, with the highly abundant U2 snRNA being the most highly represented RNA. U6 snRNA was also present at low levels, indicating that the U4/U6 particle as well as late spliceosome assembly intermediates also coprecipitated (Will and Luhrmann 2011). Consistent with our Northern blotting data, the low-abundant *TLC1* RNA that contains an Sm-binding site (Seto et al. 1999) was substantially enriched relative to other noncoding RNAs (ncRNAs), such as *SCR1*, which coprecipitated nonspecifically at low levels compared with control samples (Supplemental Table 1). Notably, the snR190 box C/D small nucleolar RNA (snoRNA) was also enriched, consistent with previously published data (Zagorski et al. 1988). Collectively, these data demonstrate the specificity and sensitivity of our analysis.

**Figure 1.**
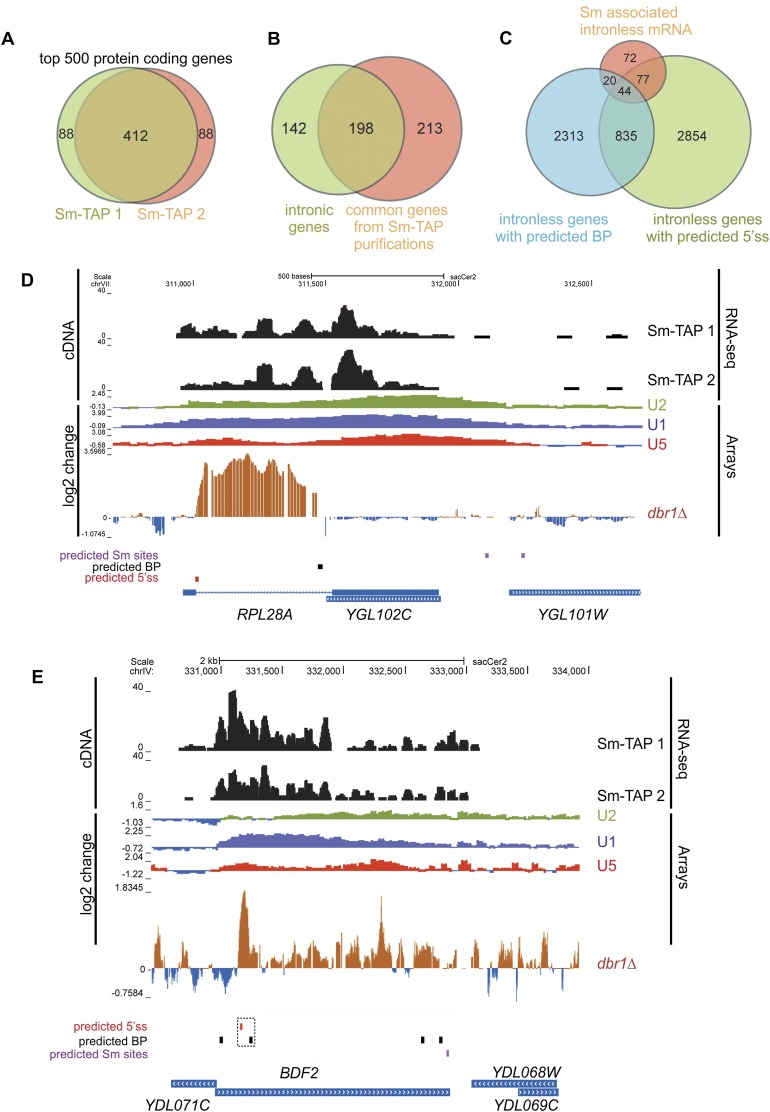
Sm proteins associate with nonintronic mRNAs. (*A*) Venn diagram showing the overlap between the top 500 mRNAs enriched in two separate SmD1-TAP experiments. (*B*) Venn diagram showing that of the 412 genes that reproducibly coprecipitated with the Sm complex, 198 contained introns, and 213 were annotated as intronless genes. The pink circle shows the 412 common genes from Sm-TAP immunoprecipitation; of these, 213 genes are nonintronic. The green circle shows 341 annotated intronic genes in *S. cerevisiae*; of these, 198 were found in Sm-TAP immunoprecipitation. (*C*) Venn diagram showing that out of all intronless genes, 835 are predicted to contain BP and 5′ss sequences in the correct order and have a potential to recruit the spliceosome. Forty-four genes with the splice site consensus were present in Sm-IP. (*D*,*E*) University of California at Santa Cruz Genome Browser outputs displaying the Sm RNA-seq results for *RPL28A*, *BDF2*, and neighboring genes. Included are the positions of the predicted 5′ss and BP splice signals. Spliceosome ChIP tiling array data (U1, U2, and U5) (Tardiff et al. 2006) and *dbr1*Δ tiling array data are indicated as “Arrays,” and log_2_ changes between mutant and wild type are shown (Tardiff et al. 2006; ENCODE Project Consortium et al. 2007).

Of the top 500 enriched mRNAs, 412 were reproducibly identified in both data sets (Fig. 1A). These included 198 intron-containing mRNAs, representing ∼60% of the known spliced mRNAs in *Saccharomyces cerevisiae* (341) (Fig. 1B). It is possible that the fraction corresponding to the remaining 40% included pre-mRNAs that are rapidly spliced and therefore underrepresented in the sequencing data. Strikingly, more than half of the 412 mRNAs identified were annotated as nonintronic (Fig. 1B). We envisage two possible explanations for this observation. First, the Sm complex could assemble directly on mRNAs independently of the spliceosome. Indeed, in silico analyses revealed 2558 potential Sm-binding sites in intronless genes and 94 in the 213 Sm-associated mRNAs (data not shown). Alternatively, this interaction could be mediated via the spliceosome, which is known to be recruited to a subset of intronless mRNAs (Kotovic et al. 2003; Tardiff et al. 2006). The relevance of these interactions, however, was not addressed, begging the question of whether these mRNAs are spliced by the spliceosome. Our in silico analyses revealed that 44 of the 213 intronless mRNAs contained canonical 5′ss (GUA[U/C/A]GU) and BP (ACUAAC[G/A/U]) sequences in the correct orientation (Fig. 1C). Three were previously described (*FBA1*, *UTH1*, and *PGI1*) (Kotovic et al. 2003), and six had a putative Sm site ([A/G]AU_4,6_G[A/G]) within 50 base pairs (bp) upstream of the 5′ss (*KAR2*, *KEM1*, *PSK1*, S*TE6*, and *YPR045C*). To validate our findings, we initially focused on the *BDF2* mRNA, as it was most likely to be targeted by the spliceosome for the following reasons: (1) *BDF2* RNA was highly enriched in both Sm-IP data sets comparable with intron-containing mRNAs (Supplemental Table 1) and has predicted 5′ss, BP, and potential 3′ss (Zhang et al. 2007) in the correct orientation (Fig. 1E). (2) U1, U2, and U5 components were found to chromatin-immunoprecipitate over the *BDF2* gene at levels comparable with a typical intron-containing gene, *RPL28A* (Fig. 1D; Tardiff et al. 2006). (3) Intron tiling array data from the *dbr1Δ* strain, where the lariat intermediate is stabilized due to the loss of debranching enzyme, have indicated the presence of a putative intron in *BDF2* mRNA (Zhang et al. 2007).

### The spliceosome endonucleolytically cleaves BDF2 mRNA

**Figure 2.**
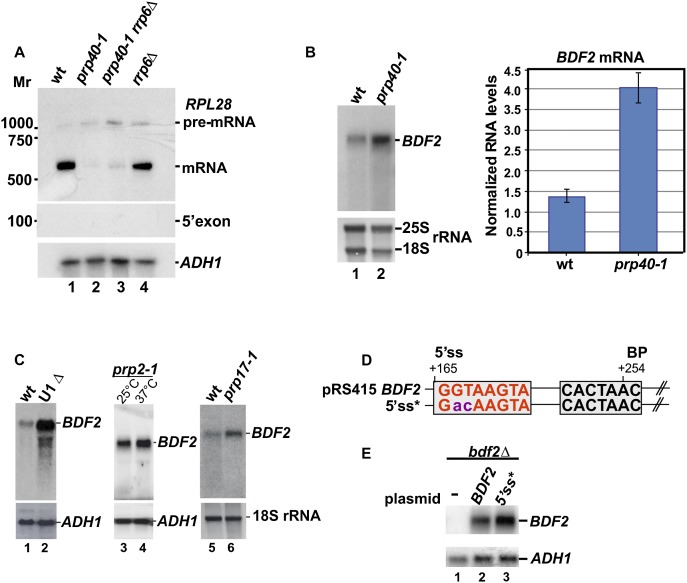
*BDF2* mRNA levels are up-regulated in the splicing mutants or upon 5′ss mutation. (*A*,*B*) Northern blot analysis on total RNA isolated from the parental strain (wild type [wt]) and *prp40-1*, *prp40-1 rrp6*Δ, and *rrp6*Δ strains. RNA was extracted 2 h after the shift to 37°C, resolved on agarose (*A*) or polyacrylamide gels (*B*), transferred to positively charged membrane, and probed for *RPL28A* (using 5′ exon-specific probe) and *BDF2* mRNAs, respectively. The positions of pre-mRNA, mature mRNA, and 5′ exon (for *RPL28A*) species are indicated. *ADH1* (*A*) or methylene blue-stained 25S and 18S rRNAs (*B*) are shown as loading controls. (*C*) Splicing mutants accumulate *BDF2* mRNA. To analyze *BDF2* mRNA levels, total RNA was extracted from U1 snRNA-depleted cells (lane *2*) and *prp2-1* (lanes *3*,*4*) and *prp17-1* (lanes *5*,*6*) mutants. Cultures were shifted to nonpermissive temperature (37°C) for 1 h (*prp2-1* and *prp17-1*) or grown in the presence of glucose for 8 h to allow for U1 depletion. (*D*) Schematic representation of the predicted splicing signals in *BDF2*. The purple nucleotides indicate the substitutions that were made in the 5′ss (red) to generate the 5′ss* construct. (*E*) Northern blot analysis of *BDF2* mRNA levels in wild-type (*BDF2*) or 5′ss mutant (5′ss*) cells.

**Figure 3.**
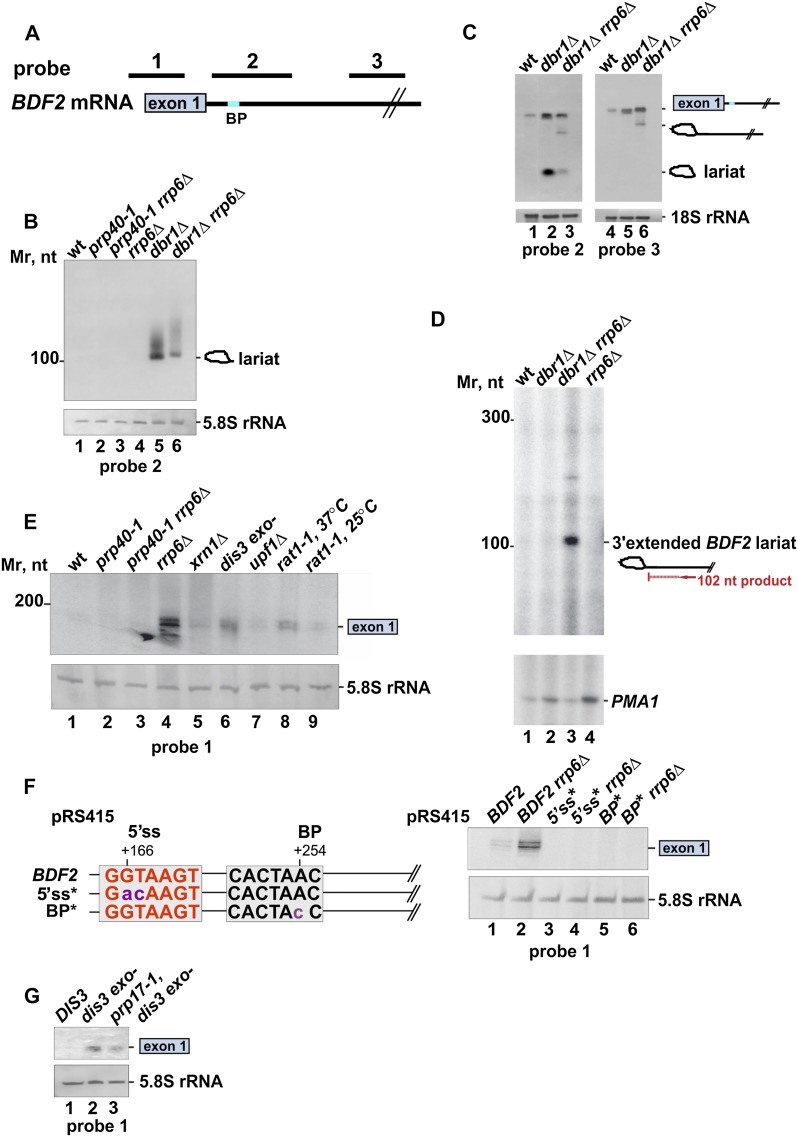
The spliceosome performs a one-step splicing reaction on *BDF2* mRNA. (*A*) Schematic representation of the *BDF2* gene. The approximate positions of probes used to detect *BDF2* mRNA and spliced products are indicated. (*B*,*C*) A *BDF2* intron–lariat accumulates in the exosome and debranching mutants. Northern blot analysis of the *BDF2* transcript was performed on RNA extracted from wild-type (wt), *prp40-1*, *prp40-1 rrp6*Δ, *rrp6*Δ, *dbr1*Δ, and *dbr1*Δ*rrp6*Δ strains. Probes 2 and 3 were used to detect the *BDF2* mRNA and lariat–intron splicing intermediates. rRNAs are shown as a loading control. A schematic representation of splicing intermediates is indicated at the side of each panel. (*D*) Primer extension analysis of the *BDF2* splicing intermediate in YF336, *dbr1*Δ, *rrp6*Δ *dbr1*Δ, and *rrp6*Δ strains. The position of the primer used in this experiment is indicated in the diagram. Primer extension products were separated on a 10% urea-PAGE. The arrow indicates the position of the primer extension product corresponding to the 3′ extended intron–lariat. Primer extension was performed on *PMA1* to control for the RNA levels. (*E*) Released *BDF2* exon 1 is degraded in the nucleus. Northern blotting was performed on RNA extracted from the wild-type, *prp40-1*, *prp40-1 rrp6*Δ, *rrp6*Δ, *upf1*Δ, *xrn1*Δ, *dis3exo*-, and *rat1-1* strains as described above. Probe 1 was used to detect the exon 1 fragment. (*F*) Mutations in the 5′ss and BP abolish the first step of *BDF2* splicing. Shown is a schematic representation of the mutations introduced to *BDF2* splice sites and Northern blot analysis of *BDF2* exon 1 in the splice site mutants. (*G*) The first step of splicing is affected in the *prp17-1* mutant. Exon 1 levels were analyzed by Northern blot. RNA was extracted from the wild-type (*DIS3*), *prp17-1*, and *prp17-1 dis3 exo*- strains.

To determine whether expression of the intronless *BDF2* mRNA is regulated by the spliceosome, we analyzed its steady-state levels in the *prp40-1* temperature-sensitive strain. Prp40 is a subunit of the U1 snRNP involved in cotranscriptional recruitment of early splicing factors to pre-mRNAs and later steps of spliceosome assembly. Previous analysis has demonstrated global down-regulation of mRNA levels in this mutant as a result of splicing defects (Albulescu et al. 2012). As expected, at the nonpermissive temperature, mRNA levels of the intron-containing gene *RPL28A* were substantially reduced (Fig. 2A). In contrast, in this strain, *BDF2* mRNA levels increased roughly threefold (Fig. 2B). Similarly, *BDF2* mRNA levels were also elevated in U1-depleted cells and *prp2-1* (Fig. 2C, lanes 1–4) and *prp42-1* (data not shown) mutants, where the first catalytic step of the splicing reaction is affected. A mutant defective in catalysis of the second transesterification reaction (*prp17-1*) (Jones et al. 1995; Noble and Guthrie 1996; Pleiss et al. 2007) also accumulated *BDF2* mRNA (Fig. 2C, lanes 5,6). Finally, a 2-base substitution introduced into the predicted 5′ss (Fig. 2D) also increased *BDF2* mRNA levels (Fig. 2E). We conclude that the spliceosome plays a direct role in down-regulating *BDF2* expression. To substantiate our results, we also performed Northern blots to measure levels of splicing intermediates in strains defective in splicing (*prp40-1*), nuclear RNA surveillance (*rrp6*Δ), and/or debranching of the intron lariat (*dbr1*Δ). Using a probe complementary to a region downstream from the predicted 5′ exon (probe 2) (Fig. 3A), we could readily detect a lariat in the *dbr1*Δ strain (Fig. 3B [lane 5], C [lane 2]). Cells lacking both Dbr1 and Rrp6 also accumulated a 3′ extended lariat intermediate (Fig. 3C [lanes 3,6], D [lane 3]). Excised exon 1, a product of the first splicing reaction, could also be detected in the *rrp6*Δ strain (Fig. 3E, lane 4), an exonuclease-defective *dis3* mutant, and a *rat1-1* temperature-sensitive mutant (Fig. 3E, lanes 6,8). We note that exon 1 fragments were heterogeneous in length, which could be the result of exosome-mediated degradation and/or low-fidelity splicing events. Deletion of the cytoplasmic exonuclease Xrn1 and the nonsense-mediated decay (NMD) factor Upf1 had no detectable effect on the stability of the exon 1 splicing products (Fig. 3E, lanes 5,7). Altogether, our data indicate that spliceosomal *BDF2* cleavage products are mainly degraded in the nucleus by both 5′–3′ (Rat1), and 3′–5′ (Rrp6 and Dis3) exonucleases. In contrast to *BDF2*, we could not detect accumulation of spliced *RPL28A* 5′exons (Fig. 2A), which is consistent with published observations for typical intronic genes (Egecioglu and Chanfreau 2011). This is most likely due to the fast kinetics of the second splicing reaction.

Accumulation of *BDF2* 5′ exon was also dependent on intact 5′ss and the BP sequences (Fig. 3F, lanes 3–6). In contrast, mutations in three predicted 3′ss had no noticeable effect on exon 1 levels or *BDF2* mRNA levels in general (Supplemental Fig. 2A; data not shown), indicating that splicing at these sites does not occur or is very inefficient. We conclude that splicing products generated using these splice elements are products of a single splicing step and are degraded by the nuclear surveillance system. This indicates that the second step of splicing in the *BDF2* mRNA is inefficient, as it was reported for the telomerase RNA (Box et al. 2008; Kannan et al. 2013). *BDF2* mRNA levels also increased in the *prp17-1* mutant, suggesting that *BDF2* can be fully spliced (Fig. 2C); however, we also noticed a reduction in exon 1 levels in the *prp17-1* strain. Consistent with previous reports, this suggests that first-step splicing is also delayed in this mutant (Sapra et al. 2008), making it difficult to determine what fraction of *BDF2* is fully spliced.

### Shortening BP–3′ss distance partially restores the second step of splicing in BDF2

The distance between the BP and 3′ss was reported to be a factor affecting efficient 3′ss selection during the second catalytic step of splicing (Luukkonen and Seraphin 1997; Box et al. 2008; Meyer et al. 2011; Kannan et al. 2013). Hence, we reasoned that the unusual long distance between these two elements in the *BDF2* mRNA might be responsible for the accumulation of the RNA species that are produced after the first step of splicing. To test whether *BDF2* mRNA has a potential to be fully spliced, we generated several *BDF2* constructs in which the distance between the BP and previously predicted 3′ss sites (Zhang et al. 2007) was shortened (Supplemental Fig. 2B, Δ1–Δ3). Most of the tested constructs failed to allow complete splicing at detectable levels even using a sensitive technique such as RT–PCR (Supplemental Fig. 2C, lanes 1,3,4,6). Intriguingly, low levels of spliced *BDF2* Δ2 product could be detected in an Rrp6 deletion strain (Supplemental Fig. 2C, lane 5), indicating that this product is degraded by the nuclear exosome. Sequencing of the PCR product revealed that this indeed represented a spliced *BDF2* mRNA in which the distal 3′ss was used (Supplemental Fig. 2D). We conclude that the previously proposed splicing signals are suboptimally positioned in *BDF2* mRNA to promote an efficient second step of splicing.

### Both a single splicing event and complete splicing contribute to regulation of BDF2 levels via SMD

**Figure 4.**
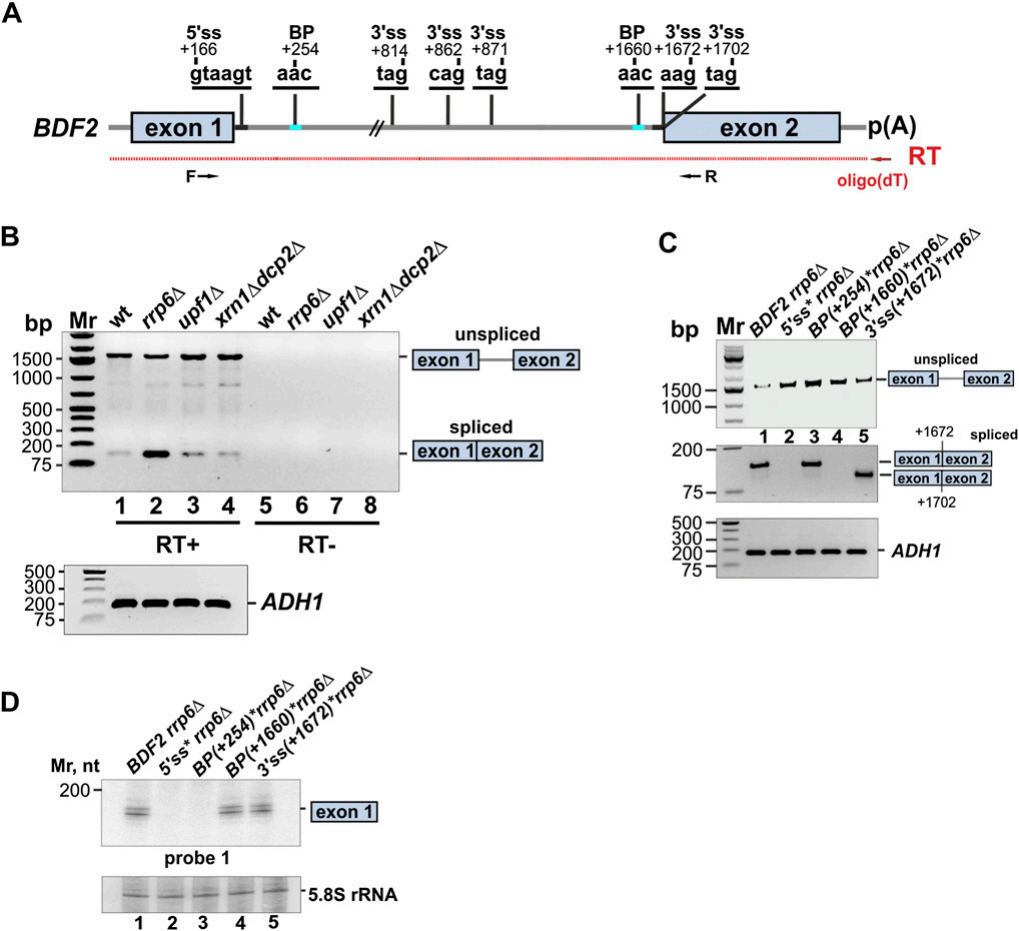
Two-step splicing also contributes to regulation of *BDF2* levels. (*A*) Shown is a schematic representation of the *BDF2* splice sites, with the position relative to ATG (+1) indicated. RT product is indicated in red, and primers used for PCR are shown as black arrows. (*B*) RT–PCR results performed on oligo[d(T)]-enriched RNA extracted from the wild-type (wt), *rrp6*Δ, *upf1*Δ, and *xrn1*Δ *dcp2*Δ strains using *BDF2* and *ADH1* primers. RT–PCR reactions without enzyme (RT−) are included as a negative control. The spliced product is indicated. (*C*) RT–PCR results performed on poly(A)-enriched RNA extracted from strains expressing the wild-type (*BDF2*) and *BDF2* 5′ss, BP (+254), BP (+1660) (changing AAC to ACC), and 3′ss (+1672) (changing AG to AA) mutants in *bdf2*Δ *rrp6*Δ. (*D*) Northern blot analysis of exon 1 levels in the strains indicated.

While this manuscript was in preparation, the Parker laboratory (Harigaya and Parker 2012) reported splicing of *BDF2* in cells lacking Xrn1 and a cytoplasmic decapping factor, Dcp2. However, the biological relevance of this splicing event had not been addressed (Harigaya and Parker 2012). Sequencing of the spliced product revealed that the 5′ss reported here and a more downstream 3′ss at nucleotide +1672 were used (Fig. 4A; Harigaya and Parker 2012). A BP consensus located 12 nucleotides (nt) upstream of this 3′ss was proposed; however, usage of this element was not experimentally validated.

We were able to detect splicing at these sites in the wild-type strain; however, no significant additional accumulation of the spliced product was in the *xrn1*Δ *dcp2*Δ double mutant (Fig. 4B, lanes 1,4). Deleting *UPF1* also did not noticeably stabilize the spliced product (Fig. 4B, lane 3), indicating that the cytoplasmic RNA surveillance machinery plays a minor role in the degradation of the spliced product. In sharp contrast, deleting *RRP6* led to an ∼4.5-fold increase in the level of the spliced product (Fig. 4B, lane 2). We conclude that SMD triggers the degradation of partially and completely spliced *BDF2* products by the nuclear surveillance machinery.

To address the importance of this two-step splicing event in the regulation of *BDF2* expression in more detail, we introduced mutations in the splice sites proposed by Harigaya and Parker (2012). *BDF2* mRNAs with mutations in the 5′ss or the BP at +1660 (Fig. 4A) were not detectably spliced, indicating that these sites are indeed used by the spliceosome. Interestingly, although the BP sequence at +254 (Fig. 4A) is clearly important for the single-step splicing event that leads to the accumulation of the exon 1 fragment in the *rrp6Δ* strain (Fig. 3F), it is not used for the two-step splicing reaction (Fig. 4C, lane 3), presumably because the distance between this BP and the downstream 3′ss is far from optimal to support splicing (>1400 nt). Surprisingly, mutating the 3′ss at +1672 did not block splicing of *BDF2*, as an alternative 3′ss was used at +1702 (Fig. 4C, lane 5). Mutations in the 5′ss or the two BPs led to a twofold to threefold increase in the *BDF2* mRNA levels (Fig. 4C, lanes 2–4). Mutation in the 3′ss at +1672 had only a modest effect on *BDF2* steady-state levels (Fig. 4C, lane 5), presumably because alternative 3′ss could still be used. In contrast, the BP at +1660 and the 3′ss at +1672 are not required for the single-step splicing event, as we could still detect accumulation of exon 1 in the *rrp6Δ* strain in cells expressing these *BDF2* mutations (Fig. 4D).

We conclude that SMD of *BDF2* involves (at least) two separate splicing events: a single cleavage event, which requires the BP at +254, and a two-step splicing event involving the BP at +1660. Both pathways use the same 5′ss, and spliced products generated by these pathways are (primarily) degraded by the nuclear surveillance machinery.

### Spliceosome recruitment to BDF2 mRNA is compromised in bdf1Δ

**Figure 5.**
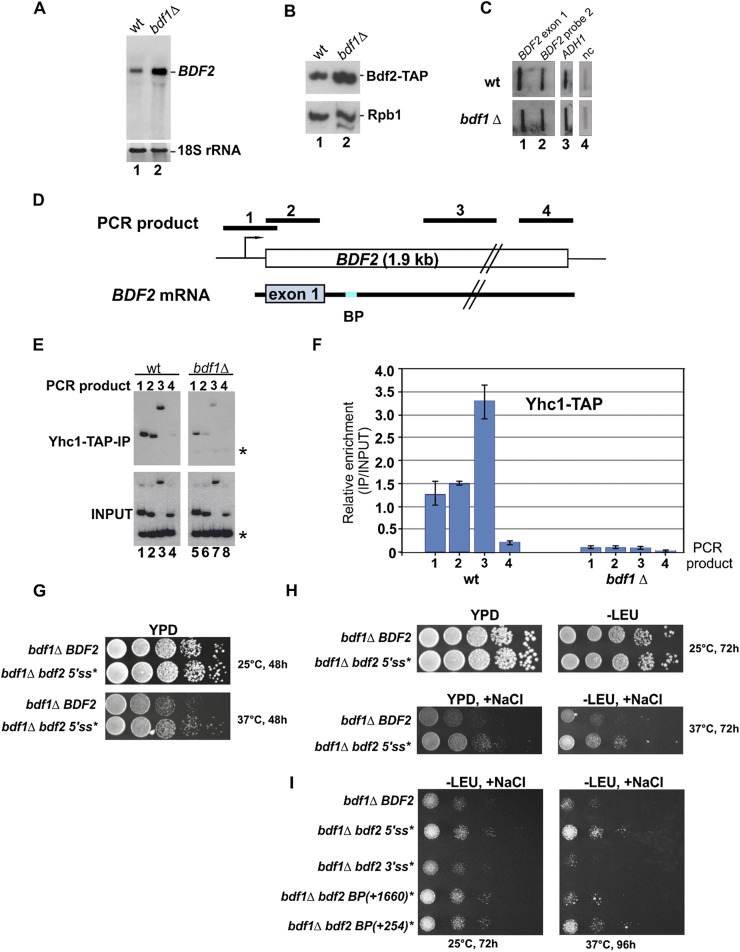
Bdf1 is required for the recruitment of the spliceosome at the *BDF2* locus. (*A*) Northern blot analysis of *BDF2* mRNA levels in the wild type (wt) and *bdf1*Δ. (*B*) Western blot analysis of Bdf2-TAP and Rpb1 (3E1 antibody from Millipore) protein levels in the wild type and *bdf1*Δ. (*C*) Deletion of Bdf1 does not noticeably affect *BDF2* transcription. Transcriptional run-on experiments were performed to measure transcription on *BDF2* in wild-type and *bdf1*Δ strains. RNA probes corresponding to *BDF2* [probe 1 (exon 1) and probe 2], *ADH1* and a nontranscribed region on chromosome V (nc) were used for detection. (*D*) Schematic representations of the *BDF2* gene and *BDF2* mRNA with the predicted 5′ exon (exon 1) and BP. Black bars show the locations of the PCR products generated in the ChIP experiments. (*E*,*F*) Deletion of *BDF1* reduces spliceosome recruitment to *BDF2*. ChIP experiments were performed in wild-type and *bdf1*Δ strains expressing TAP-tagged Yhc1 (U1C), a U1 snRNP component. U1 enrichment was measured by radioactive PCR using primers that generate the PCR fragments shown in *D*. PCR products were resolved on polyacrylamide gels and detected by autoradiography. Quantification results from four independent experiments are shown in *F*; the error bars indicate the standard error. Stars indicate position of the PCR product from a nontranscribed region on the chromosome. (*G–I*) Mutating *BDF2* 5′ss and BPs suppresses *bdf1Δ* salt- and temperature-sensitive phenotypes. The *bdf1Δ* strains expressing either *BDF2* or *bdf2* 5′ss, 3′ss, and BP mutants were grown in liquid medium until OD(*A*_600_) reached 0.5. To assay for growth, 5-μL aliquots of 10-fold serial dilutions were spotted onto YPD and −LEU medium in the presence or absence of 0.6 M NaCl.

Bdf2 and the closely related Bdf1 both bind to acetylated histones, albeit with different specificities (Matangkasombut and Buratowski 2003). These genes are genetically redundant, as only one of the two genes is necessary and sufficient for cell viability. Deletion of both genes is lethal (Matangkasombut et al. 2000). Interestingly, deletion of *BDF1* resulted in an approximately threefold increase in the levels of *BDF2* mRNA (Fig. 5A; Fu et al. 2013), similar to what we observed in the splicing mutants (Fig. 2), and a twofold increase in Bdf2 protein levels (Fig. 5B). Although Bdf1 binds to a *BDF2* promoter element (Durant and Pugh 2007; Fu et al. 2013), nuclear run-on experiments demonstrated that deletion of *BDF1* did not noticeably affect *BDF2* transcription (Fig. 5C), ruling out that Bdf1 regulates Bdf2 at the transcriptional level. Deletion *of BDF1* is known to reduce splicing of a subset of intron-containing transcripts (Albulescu et al. 2012), but the effect of *BDF1* deletion on intronless mRNA levels was not reported. This prompted us to investigate whether Bdf1 is required for spliceosome recruitment to *BDF2*. ChIP assays revealed a substantial U1 snRNP enrichment over the *BDF2* gene, in agreement with previously published data (Fig. 5D,E [lanes 1–4], F; Tardiff et al. 2006). Strikingly, deleting *BDF1* dramatically reduced the U1 binding to *BDF2* (Fig. 5E [lanes 5–8], F). Similarly, deletion of *BDF1* was reported to result in compromised U1 recruitment to several intron-containing genes, implying that Bdf1 might play a general role in splicing (Albulescu et al. 2012). We conclude that *BDF1* down-regulates *BDF2* mRNA levels by stimulating splicing of *BDF2*.

### Disrupting BDF2 splicing suppresses bdf1Δ temperature- and salt-sensitive phenotypes

To address the biological significance of regulation of *BDF2* mRNA levels via SMD, we used a genetic approach that relied on growth defects observed in the *bdf1Δ* strain. Overexpression of *BDF2* was recently shown to suppress *bdf1Δ* temperature- and salt-sensitive phenotypes in a dosage-dependent manner (Matangkasombut et al. 2000; Fu et al. 2013). We asked whether completely blocking splicing of *BDF2* by mutating the 5′ss or blocking two-step splicing by mutating the BP at +1660 (see Fig. 4A) could at least partially suppress *bdf1Δ* growth defects. Judging from the number of colonies in each dilution and the colony size, it can be concluded that at the nonpermissive temperature (37°C), *bdf1Δ* strains expressing *BDF2* mRNAs with mutated 5′ss grew better compared with the *bdf1Δ* cells expressing wild-type *BDF2* (approximately eightfold to 10-fold) (Fig. 5G). Moreover, completely blocking *BDF2* splicing substantially enhanced the cells' resistance (∼30-fold to 50-fold compared with *bdf1Δ BDF2*) to a combination of high temperature and high salt concentrations (0.6 M NaCl) (Fig. 5H). Finally, mutating the BPs also improved growth in high salt conditions (Fig. 5I). Collectively, these results demonstrate that even a relatively small change in *BDF2* mRNA and protein levels can dramatically affect a cell's resistance to certain stress conditions, underscoring the impact that SMD can have on cell fitness.

### SMD may regulate expression of ∼1% of the intronless genes

Several recent studies have described interactions between the spliceosome and mRNAs that were not known to contain introns (Kotovic et al. 2003; Moore et al. 2006; Tardiff et al. 2006; Harigaya and Parker 2012). Our bioinformatics analysis indicates that >800 genes encoding intronless mRNA have canonical 5′ss and BP sequences in the correct orientation (Fig. 1C), suggesting that the spliceosome could potentially target and regulate expression of hundreds of intronless mRNAs. To test this possibility, we performed RNA-seq analyses on rRNA-depleted RNA extracted from a wild-type strain and the temperature-sensitive *prp40-1* and *rrp6*Δ mutants.

Based on our analysis of *BDF2* mRNA, we reasoned that splicing products derived from intronless genes would be stabilized upon deleting Rrp6, whereas unspliced or full-length transcripts would accumulate in the *prp40-1* mutant. RNA was extracted from these strains 2 h after the shift to the nonpermissive temperature. This was sufficient to detect accumulation of pre-mRNAs (*prp40-1*) and 3′ extended RNA species (*rrp6*Δ) (Supplemental Fig. 3). As expected, compared with the parental strain, the levels of intron-containing mRNAs—in particular, ribosomal protein mRNAs—were substantially reduced in the *prp40-1* strain (Supplemental Fig. 4A). This global reduction of spliced mRNAs in the *prp40-1* mutant was likely the result of degradation of pre-mRNAs that accumulated in this mutant (Bousquet-Antonelli et al. 2000; Hilleren and Parker 2003). Despite this, the *prp40-1* data set contained a higher number of cDNAs that mapped to introns (Supplemental Fig. 4B), indicative of pre-mRNA accumulation due to defective splicing in this strain.

**Figure 6.**
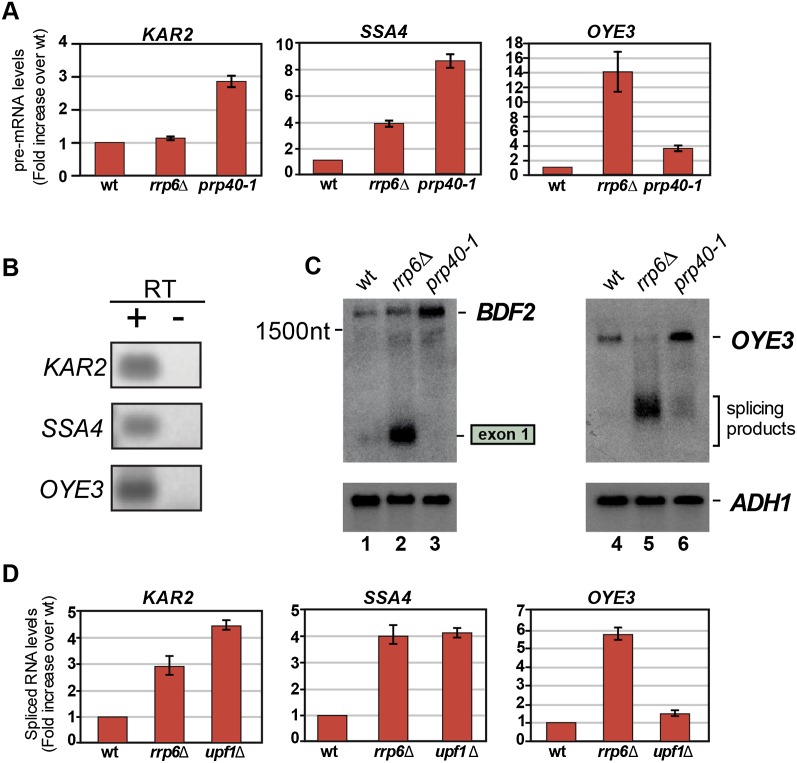
Other intronless mRNAs might be targeted by SMD. (*A*) *KAR2*, *SSA4*, and *OYE3* transcript levels increase at least threefold in the *prp40-1* mutant. To validate the RNA-seq data, mRNA levels of the transcripts predicted to be targeted by SMD was measured by qRT–PCR. Error bars indicate standard deviations. (*B*) End point RT–PCR using oligonucleotides that span exon junctions confirms splicing of the *KAR2*, *SSA4*, and *OYE3* transcripts. (*C*) *OYE3* spliced products accumulate in the *rrp6*Δ strain. Spliced and unspliced *OYE3* products were detected by Northern blot using a probe spanning the predicted intron. The *BDF2* exon 1 probe was used as a positive control. (*D*) Spliced products are stabilized in exosome (*rrp6*Δ) and (*upf1*Δ) mutants. RNA from the RNA-seq experiments was subjected to qRT–PCR using oligonucleotides that span exon junctions. The data were normalized to the *PPM2* reference gene. The *Y*-axis indicates the fold increase in the spliced RNA levels over the levels in the parental strain. Error bars indicate standard deviations.

Remarkably, in sharp contrast to intron-containing mRNAs, a large number of intronless mRNAs were up-regulated in the *prp40-1* mutant, indicative of a potential regulation by SMD (Supplemental Fig. 4A). However, we cannot rule out a possibility that increase in the levels of some of these intronless mRNAs in the *prp40-1* mutant is the result of pleiotropic effects. Compared with the parental strain, levels of 197 intronless transcripts increased at least threefold in the *prp40-1* mutant, and these were significantly enriched for genes involved in the unfolded protein response (*P*-value < 0.001). Twenty-four of 197 (∼12%) contained predicted 5′ss and BPs in the right orientation, and 16 (∼8%) were also enriched in the Sm-IP data (Supplemental Fig. 4C). Our analyses identified four transcripts (*OYE3*, *SSA4*, *KAR2*, and *BDF2*) that we predicted were most likely to be SMD targets: They reproducibly coprecipitated with the Sm proteins and contained splice signals in the correct orientation, and their steady-state levels increased at least threefold in the *prp40-1* mutant (RNA-seq and quantitative RT–PCR [qRT–PCR]) (Fig. 6A; Supplemental Fig. 4C).

Because SMD requires the activity of the nuclear RNA surveillance machinery, we looked for canonical splice junctions (GU/AG) in the sequencing data. The TopHat program (Kim and Salzberg 2011) identified splice junctions in the vast majority of known intronic mRNAs (79% [wild type] to 88% [*rrp6*Δ]) (Supplemental Fig. 5A). Splice junctions were also identified in 0.45% (wild type) to 1.1% (*rrp6*Δ) of mRNAs that are not known to contain introns. Based on these data, we predict that at least 1% of the intronless mRNAs might be subjected to SMD. For the vast majority of these transcripts, splicing would generate premature stop codons, and any spliced products would presumably be degraded. Indeed, in agreement with the unstable nature of SMD products, more splice junctions were identified in the *rrp6*Δ data set in addition to a higher number of reads mapped to protein-coding genes (Supplemental Fig. 5A). This indicates at least a partial stabilization of spliced products in the absence of Rrp6. Fewer splice junctions were recovered from the *prp40-1* data set, indicating that many of the identified junctions originated from a splicing event (Supplemental Fig. 5A).

A total of 227 canonical splice junctions were found in 49 transcripts, including *SSA4*, *KAR2*, and *OYE3* (Supplemental Table 2; Supplemental Fig. 5B), and 22 transcripts contained a BP consensus sequence within the predicted intron (Supplemental Table 3). Notably, splicing of *OYE3* at the identified splice sites would generate an in-frame deletion (Supplemental Fig. 6). However, as with *BDF2*, splicing could also occur at an alternative downstream 3′ss, generating an out-of-frame product (Supplemental Fig. 6). Unfortunately, we did not find any *BDF2* splice junctions in the RNA-seq data, but this could be due to the relatively low read coverage over *BDF2*. We did observe a modest increase in reads covering the predicted first exon in the *rrp6*Δ sequencing data (Supplemental Fig. 7), consistent with at least a partial stabilization of this product in the absence of Rrp6 (Fig. 2B).

A subset of the genes contained putative introns in the 5′ untranslated region (UTR) (*CPA2*, *GCY1*, *PIR3, SBH2*, *TBC3*, *YGR210C*, and *YRO2*) or 3′ UTR (*MSL5*, *RPL41A*, *QDR2*, and *YOR097C*). Splicing of these mRNAs is therefore not expected to alter the reading frame (Supplemental Table 2) but could potentially affect mRNA stability and/or translation efficiency.

Splicing of *SSA4*, *OYE3*, and *KAR2* was confirmed by RT–PCR and sequencing of PCR products (Fig. 6B,D; data not shown), and a strong accumulation of *OYE3* spliced products could be detected in the *rrp6*Δ strain by Northern blot (Fig. 6C). Interestingly, spliced mRNA products could be (partially) stabilized by deleting Rrp6 or Upf1, indicating that, in these cases, both the nuclear exosome and cytoplasmic surveillance machineries contribute to their degradation (Fig. 6D). Because not all of the factors involved in cytoplasmic mRNA decay were analyzed here, it is possible that the actual percentage of intronless genes regulated by SMD is higher than we predicted (1%).

We conclude that, in addition to *BDF2*, SMD also regulates expression of several other intronless genes.

## Discussion

The Sm proteins play crucial roles in pre-mRNA splicing and exert their function by binding several spliceosomal snRNAs. We tandem affinity-purified RNAs associated with the Sm complex to identify novel Sm/splicesome targets. To our surprise, more than half of the top 500 recovered mRNAs were not known to contain introns. Interactions between spliceosome components and nonintronic RNAs have been reported by other groups (Kotovic et al. 2003; Moore et al. 2006; Tardiff et al. 2006; Harigaya and Parker 2012). However, the biological relevance of these interactions remained unclear. Intriguingly, recent studies have shown that the spliceosome is also used for purposes other than removing intron sequences. In particular, it has been reported that fission yeast uses (L)Sm proteins and the spliceosome to mature the 3′ end of the telomerase RNA, involving a single spliceosome-dependent cleavage step (Box et al. 2008; Tang et al. 2012). This encouraged us to investigate the association of the spliceosome with intronless transcripts in more detail. Our bioinformatics analyses show that many intronless mRNAs contain canonical splice signals in the correct orientation and therefore have the potential to recruit the splicing machinery.

To investigate the potential function of the spliceosome on intronless genes, we focused on *BDF2*, a gene encoding a bromodomain transcription factor, where we and others (Tardiff et al. 2006) observed recruitment of the spliceosome. Using yeast strains defective in nuclear RNA surveillance and pre-mRNA splicing, we discovered that splicing of the *BDF2* mRNA led to an approximately threefold reduction in *BDF2* mRNA levels, and the resulting splicing products were rapidly degraded. This phenomenon, which we refer to as SMD, down-regulates mRNA expression as opposed to a well-documented positive outcome of conventional splicing and could potentially play an important role in down-regulating transcript levels. Indeed, the 5′ss and BP consensus sequences are conserved in genes homologous to *BDF2* in related yeast species (Supplemental Fig. 8). Transcriptome sequencing of splicing and exosome mutants identified several splicing events on transcripts that were previously regarded not to have introns. We furthermore demonstrate that *KAR2*, *SSA4*, and *OYE3* mRNA levels are also regulated via SMD and identify dozens of other potential targets. Finally, we show that Bdf1, a Bdf2 paralog, is required for the recruitment of the spliceosome to *BDF2*, revealing a novel mechanism by which expression of paralogous genes can be regulated.

**Figure 7.**
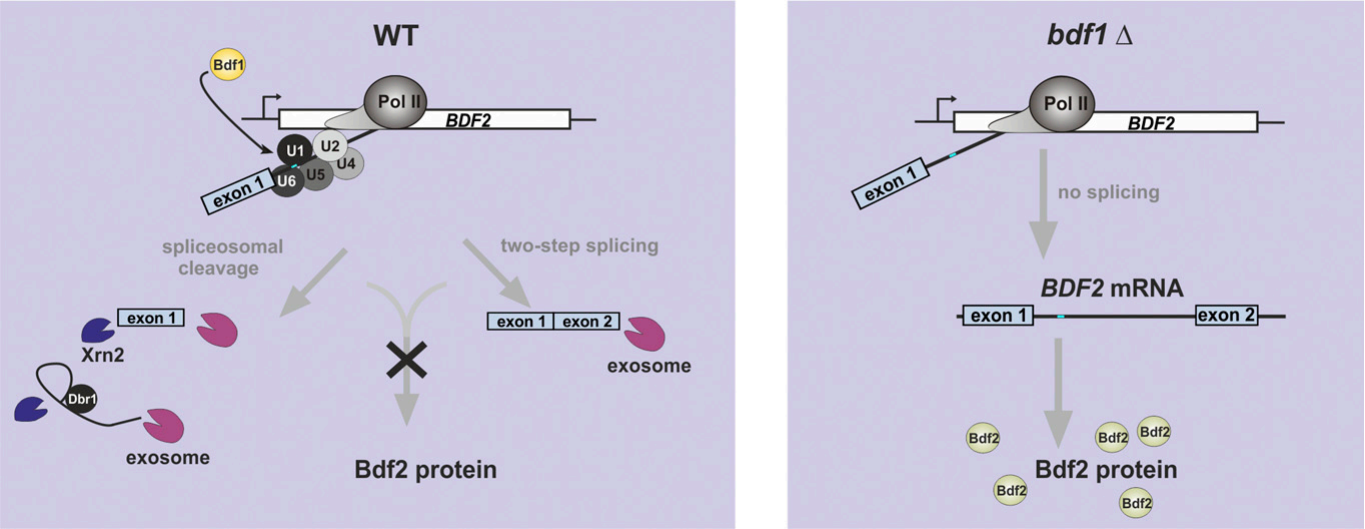
Model for the regulation of *BDF2* expression via SMD. In the presence of Bdf1, the spliceosome assembles on the *BDF2* mRNA. This leads to cleavage at the 5′ss and (1) subsequent release of exon 1 and (2) complete splicing. Splicing products are degraded by the nuclear exosome (and 5′–3′ exonuclease Rat1 may contribute as well), leading to down-regulation of Bdf2 levels. In the absence of Bdf1, the *BDF2* mRNA is not efficiently recognized by the spliceosome, and mainly nonspliced full-length *BDF2* mRNA is generated.

A growing body of evidence suggests that defective or inefficient pre-mRNA splicing leads to the production of nonfunctional and potentially toxic RNAs such as unspliced mRNA precursors as well as various splicing intermediates. These are targeted by the cellular RNA surveillance systems for destruction. The current model implicates spliceosomal components in the initial identification of defective splicing intermediates, which are discarded by the spliceosome and released to be degraded by the cellular RNA decay machineries (Houseley et al. 2006; Egecioglu and Chanfreau 2011; Parker 2012; Schmid and Jensen 2013). Unspliced RNAs are degraded by the exosome (from 3′ to 5′), Rat1/Xrn2, and Xrn1 (from 5′ to 3′) in both nuclear and cytoplasmic compartments. Our results show that *BDF2* splicing intermediates that accumulate due to delayed second step of splicing are also degraded. It was previously proposed that splicing intermediates are degraded in the cytoplasm (Hilleren and Parker 2003; Sayani et al. 2008; Mayas et al. 2010; Harigaya and Parker 2012). However, here we demonstrate that splicing products are also degraded by the nuclear exosome, as they accumulate in cells lacking Rrp6. How are these RNAs recognized by the nuclear exosome? We speculate that recognition by the nuclear surveillance machinery is coupled to the nuclear retention times of these molecules. Growing evidence suggests that splicing and surveillance are tightly connected and that the fate of pre-mRNAs is dictated by competition between degradation, splicing, and export machineries (Gudipati et al. 2012; Sayani and Chanfreau 2012). Unspliced transcripts and splicing intermediates that are efficiently exported to the cytoplasm are presumably more likely to be degraded by the cytoplasmic RNA decay machinery, whereas transcripts with relatively slow export kinetics or long nuclear retention times are (also) targeted by the nuclear degradation pathway (Sayani and Chanfreau 2012). This model also implies that slow transition kinetics from the first to the second step of splicing could render RNA more susceptible to nuclear degradation. The efficiency of the 3′ss selection depends on several factors, including the distance of 3′ss from the BP, secondary structure of the intron, and interactions with the specific protein factors (Box et al. 2008; Meyer et al. 2011). Here we demonstrate that regulation of *BDF2* by SMD can involve (at least) two different splicing events in which the same 5′ss is used but two different BP sequences (see Fig. 7). We speculate that these two BPs are competing with each other for splicing factors and that selection of the BP is stochastic. If the spliceosome uses the upstream BP (+254), then it is very unlikely that the second splicing step will take place, as the BP is more than several hundred nucleotides away from 3′ss elements (Zhang et al. 2007; Harigaya and Parker 2012). When the downstream BP is used, complete splicing can occur. Surprisingly, products generated by either pathway appear to be primarily degraded by the nuclear RNA surveillance machinery, as deletion of Rrp6 resulted in a substantial increase of partially and completely spliced products. Mutating either BP results in a twofold to threefold increase in *BDF2* steady-state levels, suggesting that both pathways contribute significantly to SMD. The selection of BP and 3′ss signals is likely the rate-limiting step in splicing of *BDF2*, and any delay presumably leads to the activation of the nuclear surveillance machinery. This model is supported by a recent study from the Baumann laboratory (Kannan et al. 2013) that focused on dissecting the mechanism of a one-step splicing reaction required for 3′ end formation of the telomerase RNA in fission yeast. This study reports that slow kinetics of the second splicing reaction due to the suboptimal positioning of the 3′ss could lead to the structural rearrangements of the spliceosome complex and subsequent discard of the intermediates. We propose that, depending on the rate of the second step of splicing, the spliceosome could either direct RNA degradation (*BDF2*) or splice pre-mRNA (conventionally spliced mRNA-encoding genes). Importantly, each of these two scenarios leads to a completely different outcome: Production of unstable aberrant RNAs negatively impacts gene expression (Fig. 7), whereas conventional splicing produces functional protein-coding mRNA.

In *S. cerevisiae*, only a small proportion of genes contains introns and encodes transcripts that are spliced. As a possible explanation for this phenomenon, it was previously proposed that introns in many genes of the budding yeast were “lost” during the course of evolution (Fink 1987). However, recent studies as well as our data show recruitment of the spliceosome coinciding with the presence of conserved splice signals on genes that are not known to be spliced. It seems unlikely that these conserved sequences were retained through evolution without any functional implication. Indeed, we observed that a number of RNAs undergo splicing, creating fully spliced but nonfunctional RNAs that are subsequently degraded via SMD. While spliced *BDF2* products are mainly targeted in the nucleus by SMD, some products of unproductive splicing can also be degraded by NMD in the cytoplasm.

It is not clear exactly how many other genes are regulated by SMD to the same degree as *BDF2*. Under the experimental conditions used, for some intronless transcripts in which we could detect splice junctions in the RNA-seq data, we could not detect a significant increase in pre-mRNA levels in splicing mutants, indicating that splicing of these mRNAs is inefficient. It is conceivable that the presence of (canonical) splice sites is sufficient to recruit the spliceosome and perhaps drive splicing to some degree, but for most transcripts, this is not sufficient to dramatically alter steady-state RNA levels. It is also possible that significant changes will only be detectable under specific growth or stress conditions. Examples of this regulation were reported in higher eukaryotes. Thus, the well-documented unproductive outcome of alternative splicing can be used to regulate the expression of specific genes by suppressing the production of a protein in the absence of the proper biological context (Lareau et al. 2007; Ni et al. 2007). For example, the expression of PTBP1 and PTBP2 factors is regulated via this mechanism in order to control their function during neuronal differentiation (Wollerton et al. 2004). In this scenario, alternative splicing results in production of a nonfunctional RNA degraded by NMD. Experiments in the laboratory are most frequently performed when cells are growing exponentially in high concentrations of glucose or galactose, conditions rarely found in nature. Under these conditions, cells devote the vast majority of their resources to the synthesis and splicing of pre-mRNAs encoding ribosomal proteins, masking the detection of less abundant or less efficient splicing events. Indeed, differential recruitment profiles of the spliceosome and degradation machineries to target substrates were reported in response to different carbon sources (Bousquet-Antonelli et al. 2000), environmental stress (Moore et al. 2006; Pleiss et al. 2007), the cell cycle (Reis and Campbell 2007; Mullen and Marzluff 2008), and meiosis (Moore et al. 2006; McPheeters et al. 2009; Cremona et al. 2011). *BDF2* expression has also been reported to be significantly affected under different stress conditions (Causton et al. 2001; Tkach et al. 2012).

Consistent with recently published work, we also observed an increase in *BDF2* mRNA levels upon deletion of another bromodomain-containing protein, *BDF1*, whose function in transcription initiation is redundant with Bdf2 (Matangkasombut et al. 2000; Durant and Pugh 2007). We reasoned that the spliceosome could potentially regulate Bdf2 function in the cell by modulating its levels and therefore analyzed recruitment of U1 snRNP in *bdf1*Δ. Indeed, our data suggest that the approximately threefold increase in *BDF2* mRNA levels (and approximately twofold increase in protein level) in *bdf1*Δ is due to the reduced recruitment of the spliceosome to *BDF2* (Fig. 7). The function of Bdf1 in regulating gene expression via splicing, in addition to its role in transcription initiation, may explain the multiple phenotypes associated with the lack of Bdf1. For example, *BDF1* deletion causes slow growth, temperature sensitivity, salt hypersensitivity, flocculence in liquid culture, sensitivity to the DNA-damaging agent MMS, poor sporulation, and inability to grow on nonfermentable carbon sources (Lygerou et al. 1994; Chua and Roeder 1995; Liu et al. 2007).

It remains to be determined whether Bdf1 is directly involved in the recruitment of the splicing machinery or whether this is a secondary effect of its function in transcription. Deletion of Bdf1 was reported to affect snRNA levels, indicating a role in transcription of these genes (Lygerou et al. 1994); however, a more recent study has questioned these findings (Albulescu et al. 2012). We favor a model in which Bdf1 plays a direct role in regulating *BDF2* expression via modulating spliceosome recruitment. The latter could be inferred from the fact that the presence of Bdf1 on the *BDF2* gene (Venters et al. 2011) has no noticeable impact on transcription (Fig. 4C). Moreover, loss of Bdf1 was previously linked with genome-wide defective splicing and impaired recruitment of the spliceosome to spliced genes, implying a general role in splicing (Albulescu et al. 2012). As regulation at the level of chromatin has recently emerged as an additional regulatory mechanism affecting splicing (de Almeida and Carmo-Fonseca 2012), it would be interesting to examine whether Bdf1 function in splicing is mediated via recognition of the acetylated histones.

In conclusion, we propose that the cooperative contribution of multiple mechanisms during RNA processing—such as recruitment of the spliceosome, the checkpoint allowing the second step of splicing, and finally, recruitment of the exosome to the defective RNAs—is important to ensure dynamic regulation of gene expression in eukaryotes.

## Materials and methods

### Yeast strains

The *S. cerevisiae* strains used in this study are listed in Supplemental Table 4. A list of the oligonucleotides used is given in Supplemental Table 5.

### Northern blotting

RNA extractions and Northern blot experiments were performed as previously described (Vasiljeva and Buratowski 2006). Gene-specific PCR-generated fragments were used as probes using the oligonucleotides listed in Supplemental Table 5.

### Purification of the Sm complex

TAP-tagged Sm complex was purified from 16 L of yeast culture essentially as described in Vasiljeva and Buratowski (2006). RNA was extracted from calmodulin-bound material and subjected to RNA-seq.

### Transcriptional run-on

Transcriptional run-on was performed as described in Birse et al. (1997). Single-stranded probes for transcriptional run-on analysis were generated by in vitro transcription using T7 or SP6 polymerase from plasmids harboring regions corresponding to different regions of *BDF2*. *BDF2*- and *ADH1*-derived fragments were generated using the primers listed in Supplemental Table 5 and were cloned into the pCR-Blunt II-TOPO vector (Invitrogen).

### ChIP experiments

ChIP procedures and quantification were performed as described (Keogh and Buratowski 2004; Kim et al. 2004). For temperature shift experiments, cells were incubated at 23°C until OD(A_600_) = 0.5, and cells were further incubated for 120 min at 37°C. The primers are listed in Supplemental Table 5.

### High-throughput RNA-seq

Procedures for cDNA library preparation and RNA-seq are described in the Supplemental Material. Raw (fastq) and processed (bedgraph and GTF files) RNA-seq data can be downloaded from the NCBI Gene Expression Omnibus repository (http://www.ncbi.nlm.nih.gov/geo, accession number GSE49966).

## References

[B1] Albulescu LO, Sabet N, Gudipati M, Stepankiw N, Bergman ZJ, Huffaker TC, Pleiss JA 2012 A quantitative, high-throughput reverse genetic screen reveals novel connections between Pre-mRNA splicing and 5′ and 3′ end transcript determinants. PLoS Genet 8: e1002530 22479188 10.1371/journal.pgen.1002530 PMC3315463

[B2] Beggs JD 2005 Lsm proteins and RNA processing. Biochem Soc Trans 33: 433–438 15916535 10.1042/BST0330433

[B3] Birse CE, Lee BA, Hansen K, Proudfoot NJ 1997 Transcriptional termination signals for RNA polymerase II in fission yeast. EMBO J 16: 3633–3643 9218804 10.1093/emboj/16.12.3633 PMC1169987

[B4] Bousquet-Antonelli C, Presutti C, Tollervey D 2000 Identification of a regulated pathway for nuclear pre-mRNA turnover. Cell 102: 765–775 11030620 10.1016/s0092-8674(00)00065-9

[B5] Box JA, Bunch JT, Tang W, Baumann P 2008 Spliceosomal cleavage generates the 3′ end of telomerase RNA. Nature 456: 910–914 19052544 10.1038/nature07584

[B6] Carrillo Oesterreich F, Bieberstein N, Neugebauer KM 2011 Pause locally, splice globally. Trends Cell Biol 21: 328–335 21530266 10.1016/j.tcb.2011.03.002

[B7] Causton HC, Ren B, Koh SS, Harbison CT, Kanin E, Jennings EG, Lee TI, True HL, Lander ES, Young RA 2001 Remodeling of yeast genome expression in response to environmental changes. Mol Biol Cell 12: 323–337 11179418 10.1091/mbc.12.2.323 PMC30946

[B8] Chua P, Roeder GS 1995 Bdf1, a yeast chromosomal protein required for sporulation. Mol Cell Biol 15: 3685–3696 7791775 10.1128/mcb.15.7.3685 PMC230606

[B9] Cremona N, Potter K, Wise JA 2011 A meiotic gene regulatory cascade driven by alternative fates for newly synthesized transcripts. Mol Biol Cell 22: 66–77 21148298 10.1091/mbc.E10-05-0448 PMC3016978

[B10] de Almeida SF, Carmo-Fonseca M 2012 Design principles of interconnections between chromatin and pre-mRNA splicing. Trends Biochem Sci 37: 248–253 22398209 10.1016/j.tibs.2012.02.002

[B11] Durant M, Pugh BF 2007 NuA4-directed chromatin transactions throughout the *Saccharomyces cerevisiae* genome. Mol Cell Biol 27: 5327–5335 17526728 10.1128/MCB.00468-07 PMC1952100

[B12] Egecioglu DE, Chanfreau G 2011 Proofreading and spellchecking: A two-tier strategy for pre-mRNA splicing quality control. RNA 17: 383–389 21205840 10.1261/rna.2454711 PMC3039138

[B13] ENCODE Project Consortium, Birney E, Stamatoyannopoulos JA, Dutta A, Guigo R, Gingeras TR, Margulies EH, Weng Z, Snyder M, Dermitzakis ET, 2007 Identification and analysis of functional elements in 1% of the human genome by the ENCODE pilot project. Nature 447: 799–816 17571346 10.1038/nature05874 PMC2212820

[B14] Fink GR 1987 Pseudogenes in yeast? Cell 49: 5–6 3549000 10.1016/0092-8674(87)90746-x

[B15] Fu J, Hou J, Liu L, Chen L, Wang M, Shen Y, Zhang Z, Bao X 2013 Interplay between BDF1 and BDF2 and their roles in regulating the yeast salt stress response. FEBS J 280: 1991–2001 23452060 10.1111/febs.12219

[B16] Gudipati RK, Xu Z, Lebreton A, Seraphin B, Steinmetz LM, Jacquier A, Libri D 2012 Extensive degradation of RNA precursors by the exosome in wild-type cells. Mol Cell 48: 409–421 23000176 10.1016/j.molcel.2012.08.018 PMC3496076

[B17] Harigaya Y, Parker R 2012 Global analysis of mRNA decay intermediates in *Saccharomyces cerevisiae*. Proc Natl Acad Sci 109: 11764–11769 22752303 10.1073/pnas.1119741109 PMC3406813

[B18] Hilleren PJ, Parker R 2003 Cytoplasmic degradation of splice-defective pre-mRNAs and intermediates. Mol Cell 12: 1453–1465 14690599 10.1016/s1097-2765(03)00488-x

[B19] Houseley J, LaCava J, Tollervey D 2006 RNA-quality control by the exosome. Nat Rev Mol Cell Biol 7: 529–539 16829983 10.1038/nrm1964

[B20] Jones MH, Frank DN, Guthrie C 1995 Characterization and functional ordering of Slu7p and Prp17p during the second step of pre-mRNA splicing in yeast. Proc Natl Acad Sci 92: 9687–9691 7568198 10.1073/pnas.92.21.9687 PMC40867

[B21] Kannan R, Hartnett S, Voelker RB, Berglund JA, Staley JP, Baumann P 2013 Intronic sequence elements impede exon ligation and trigger a discard pathway that yields functional telomerase RNA in fission yeast. Genes Dev 27: 627–638 23468430 10.1101/gad.212738.112 PMC3613610

[B22] Keogh MC, Buratowski S 2004 Using chromatin immunoprecipitation to map cotranscriptional mRNA processing in *Saccharomyces cerevisiae*. Methods Mol Biol 257: 1–16 14769992 10.1385/1-59259-750-5:001

[B23] Kim D, Salzberg SL 2011 TopHat-Fusion: An algorithm for discovery of novel fusion transcripts. Genome Biol 12: R72 21835007 10.1186/gb-2011-12-8-r72 PMC3245612

[B24] Kim M, Ahn SH, Krogan NJ, Greenblatt JF, Buratowski S 2004 Transitions in RNA polymerase II elongation complexes at the 3′ ends of genes. EMBO J 23: 354–364 14739930 10.1038/sj.emboj.7600053 PMC1271760

[B25] Kotovic KM, Lockshon D, Boric L, Neugebauer KM 2003 Cotranscriptional recruitment of the U1 snRNP to intron-containing genes in yeast. Mol Cell Biol 23: 5768–5779 12897147 10.1128/MCB.23.16.5768-5779.2003 PMC166328

[B26] Lareau LF, Inada M, Green RE, Wengrod JC, Brenner SE 2007 Unproductive splicing of SR genes associated with highly conserved and ultraconserved DNA elements. Nature 446: 926–929 17361132 10.1038/nature05676

[B27] Liu X, Zhang X, Wang C, Liu L, Lei M, Bao X 2007 Genetic and comparative transcriptome analysis of bromodomain factor 1 in the salt stress response of *Saccharomyces cerevisiae*. Curr Microbiol 54: 325–330 17334841 10.1007/s00284-006-0525-4

[B28] Luukkonen BG, Seraphin B 1997 The role of branchpoint–3′ splice site spacing and interaction between intron terminal nucleotides in 3′ splice site selection in *Saccharomyces cerevisiae*. EMBO J 16: 779–792 9049307 10.1093/emboj/16.4.779 PMC1169679

[B29] Lygerou Z, Conesa C, Lesage P, Swanson RN, Ruet A, Carlson M, Sentenac A, Seraphin B 1994 The yeast BDF1 gene encodes a transcription factor involved in the expression of a broad class of genes including snRNAs. Nucleic Acids Res 22: 5332–5340 7816623 10.1093/nar/22.24.5332 PMC332079

[B30] Matangkasombut O, Buratowski S 2003 Different sensitivities of bromodomain factors 1 and 2 to histone H4 acetylation. Mol Cell 11: 353–363 12620224 10.1016/s1097-2765(03)00033-9

[B31] Matangkasombut O, Buratowski RM, Swilling NW, Buratowski S 2000 Bromodomain factor 1 corresponds to a missing piece of yeast TFIID. Genes Dev 14: 951–962 10783167 PMC316539

[B32] Mayas RM, Maita H, Semlow DR, Staley JP 2010 Spliceosome discards intermediates via the DEAH box ATPase Prp43p. Proc Natl Acad Sci 107: 10020–10025 20463285 10.1073/pnas.0906022107 PMC2890470

[B33] McPheeters DS, Cremona N, Sunder S, Chen HM, Averbeck N, Leatherwood J, Wise JA 2009 A complex gene regulatory mechanism that operates at the nexus of multiple RNA processing decisions. Nat Struct Mol Biol 16: 255–264 19198588 10.1038/nsmb.1556 PMC2776722

[B34] Meyer M, Plass M, Perez-Valle J, Eyras E, Vilardell J 2011 Deciphering 3′ss selection in the yeast genome reveals an RNA thermosensor that mediates alternative splicing. Mol Cell 43: 1033–1039 21925391 10.1016/j.molcel.2011.07.030

[B35] Moore MJ, Proudfoot NJ 2009 Pre-mRNA processing reaches back to transcription and ahead to translation. Cell 136: 688–700 19239889 10.1016/j.cell.2009.02.001

[B36] Moore MJ, Schwartzfarb EM, Silver PA, Yu MC 2006 Differential recruitment of the splicing machinery during transcription predicts genome-wide patterns of mRNA splicing. Mol Cell 24: 903–915 17189192 10.1016/j.molcel.2006.12.006

[B37] Morris DP, Greenleaf AL 2000 The splicing factor, Prp40, binds the phosphorylated carboxyl-terminal domain of RNA polymerase II. J Biol Chem 275: 39935–39943 10978320 10.1074/jbc.M004118200

[B38] Mullen TE, Marzluff WF 2008 Degradation of histone mRNA requires oligouridylation followed by decapping and simultaneous degradation of the mRNA both 5′ to 3′ and 3′ to 5′. Genes Dev 22: 50–65 18172165 10.1101/gad.1622708 PMC2151014

[B39] Ni JZ, Grate L, Donohue JP, Preston C, Nobida N, O'Brien G, Shiue L, Clark TA, Blume JE, Ares M Jr 2007 Ultraconserved elements are associated with homeostatic control of splicing regulators by alternative splicing and nonsense-mediated decay. Genes Dev 21: 708–718 17369403 10.1101/gad.1525507 PMC1820944

[B40] Noble SM, Guthrie C 1996 Identification of novel genes required for yeast pre-mRNA splicing by means of cold-sensitive mutations. Genetics 143: 67–80 8722763 10.1093/genetics/143.1.67 PMC1207296

[B41] Parker R 2012 RNA degradation in *Saccharomyces cerevisae*. Genetics 191: 671–702 22785621 10.1534/genetics.111.137265 PMC3389967

[B42] Pleiss JA, Whitworth GB, Bergkessel M, Guthrie C 2007 Rapid, transcript-specific changes in splicing in response to environmental stress. Mol Cell 27: 928–937 17889666 10.1016/j.molcel.2007.07.018 PMC2081968

[B43] Reis CC, Campbell JL 2007 Contribution of Trf4/5 and the nuclear exosome to genome stability through regulation of histone mRNA levels in *Saccharomyces cerevisiae*. Genetics 175: 993–1010 17179095 10.1534/genetics.106.065987 PMC1840065

[B44] Sapra AK, Khandelia P, Vijayraghavan U 2008 The splicing factor Prp17 interacts with the U2, U5 and U6 snRNPs and associates with the spliceosome pre- and post-catalysis. Biochem J 416: 365–374 18691155 10.1042/BJ20081195

[B45] Sayani S, Chanfreau GF 2012 Sequential RNA degradation pathways provide a fail-safe mechanism to limit the accumulation of unspliced transcripts in *Saccharomyces cerevisiae*. RNA 18: 1563–1572 22753783 10.1261/rna.033779.112 PMC3404376

[B46] Sayani S, Janis M, Lee CY, Toesca I, Chanfreau GF 2008 Widespread impact of nonsense-mediated mRNA decay on the yeast intronome. Mol Cell 31: 360–370 18691968 10.1016/j.molcel.2008.07.005 PMC2600495

[B47] Schmid M, Jensen TH 2013 Transcription-associated quality control of mRNP. Biochim Biophys Acta 1829: 158–168 22982197 10.1016/j.bbagrm.2012.08.012

[B48] Seto AG, Zaug AJ, Sobel SG, Wolin SL, Cech TR 1999 *Saccharomyces cerevisiae* telomerase is an Sm small nuclear ribonucleoprotein particle. Nature 401: 177–180 10490028 10.1038/43694

[B49] Tang W, Kannan R, Blanchette M, Baumann P 2012 Telomerase RNA biogenesis involves sequential binding by Sm and Lsm complexes. Nature 484: 260–264 22446625 10.1038/nature10924 PMC3326189

[B50] Tardiff DF, Lacadie SA, Rosbash M 2006 A genome-wide analysis indicates that yeast pre-mRNA splicing is predominantly posttranscriptional. Mol Cell 24: 917–929 17189193 10.1016/j.molcel.2006.12.002 PMC1828117

[B51] Tkach JM, Yimit A, Lee AY, Riffle M, Costanzo M, Jaschob D, Hendry JA, Ou J, Moffat J, Boone C, 2012 Dissecting DNA damage response pathways by analysing protein localization and abundance changes during DNA replication stress. Nat Cell Biol 14: 966–976 22842922 10.1038/ncb2549 PMC3434236

[B52] Vasiljeva L, Buratowski S 2006 Nrd1 interacts with the nuclear exosome for 3′ processing of RNA polymerase II transcripts. Mol Cell 21: 239–248 16427013 10.1016/j.molcel.2005.11.028

[B53] Venters BJ, Wachi S, Mavrich TN, Andersen BE, Jena P, Sinnamon AJ, Jain P, Rolleri NS, Jiang C, Hemeryck-Walsh C, 2011 A comprehensive genomic binding map of gene and chromatin regulatory proteins in *Saccharomyces*. Mol Cell 41: 480–492 21329885 10.1016/j.molcel.2011.01.015 PMC3057419

[B54] Wahl MC, Will CL, Luhrmann R 2009 The spliceosome: Design principles of a dynamic RNP machine. Cell 136: 701–718 19239890 10.1016/j.cell.2009.02.009

[B55] Will CL, Luhrmann R 2011 Spliceosome structure and function. Cold Spring Harb Perspect Biol 3: a003707 21441581 10.1101/cshperspect.a003707 PMC3119917

[B56] Wollerton MC, Gooding C, Wagner EJ, Garcia-Blanco MA, Smith CW 2004 Autoregulation of polypyrimidine tract binding protein by alternative splicing leading to nonsense-mediated decay. Mol Cell 13: 91–100 14731397 10.1016/s1097-2765(03)00502-1

[B57] Yoshikawa K, Tanaka T, Ida Y, Furusawa C, Hirasawa T, Shimizu H 2011 Comprehensive phenotypic analysis of single-gene deletion and overexpression strains of *Saccharomyces cerevisiae*. Yeast 28: 349–361 21341307 10.1002/yea.1843

[B58] Zagorski J, Tollervey D, Fournier MJ 1988 Characterization of an SNR gene locus in *Saccharomyces cerevisiae* that specifies both dispensible and essential small nuclear RNAs. Mol Cell Biol 8: 3282–3290 2850487 10.1128/mcb.8.8.3282-3290.1988 PMC363561

[B59] Zhang D, Abovich N, Rosbash M 2001 A biochemical function for the Sm complex. Mol Cell 7: 319–329 11239461 10.1016/s1097-2765(01)00180-0

[B60] Zhang Z, Hesselberth JR, Fields S 2007 Genome-wide identification of spliced introns using a tiling microarray. Genome Res 17: 503–509 17351133 10.1101/gr.6049107 PMC1832097

